# Data set on training assistance and the performance of small and medium enterprises in Lagos, Nigeria

**DOI:** 10.1016/j.dib.2018.07.023

**Published:** 2018-07-19

**Authors:** Fred Ojochide Peter, Patrick Oladele, Omotayo Adegbuyi, Ayodele Maxwell Olokundun, Adeshola Oluwaseyi Peter, Augusta Bosede Amaihian, Ayodotun Stephen Ibidunni, Odunayo Paul Salau

**Affiliations:** a Department of Business Management, Covenant University, Ota, Ogun State, Nigeria; b Faculty of Management Sciences, Ekiti State University, Ado-Ekiti, Nigeria

## Abstract

Human capital is considered a major asset of any business. This is even more vital in a knowledge-driven economy, where non-visible factors and services are of increasingly significant. The focus of this study was to determine the impact of training assistance on the performance of SMEs in Lagos, Nigeria. Only few studies have investigated how training support programmes by government facilitate the performance of SMEs under Small and Medium Enterprises Agency of Nigeria (SMEDAN) in Nigeria. Descriptive research method was adopted for this study. Statistical Package for Social Sciences (SPSS 22) was used to analyse 203 copies of questionnaire retrieved. The reliability and validity of research instruments was also established. This data set indicated that training and development can facilitate innovative processes among SMEs owners to create economic, social values and remain globally competitive.

**Specifications Table**

**Table:** 

Subject area	Management
More Specific Subject Area	Small Business and Entrepreneurship
Type of Data	Table
How Data was Acquired	*Field survey*
Data format	Raw, analysed, Inferential statistical data
Experimental Factors	This study used Statistical Package for Social Sciences (SPSS version20). Descriptive research design approach was used to analyse the data obtained from copies questionnaire.
Experimental features	Data was obtained from structured questionnaires administered to Small and Medium Enterprises(SMEs) Lago state, Nigerian who have benefited from government training assistance under the supervision of Small and Medium Enterprises Agency of Nigeria (SMEDAN).
Data source location	SMEs Operators in Lagos, Nigeria
Data Accessibility	Data is included in this article

**Value of the data**

• These data present information on training assistance as regards the performance of SMEs. This is important considering the fact that developing the right skill set implies improved capacity to deliver superior value to customer.
• This data shows the significance of providing adequate training assistance particularly in conjunction with incubator system for Small and Medium Enterprises (SMEs), its impact and valuable contributions in enhancing the innovative performance.
• The results can facilitate innovative processes among SMEs owners to create both economic and social values and remain competitive.

## 1 Data

The data for this research was gathered from SMEs owners/managers in Lagos, who have benefited from government training assistance. A total of two hundred (203) copies of questionnaire were analysed. Descriptive statistics and regression analysis was used to facilitate the analysis of data obtained. It shows the degree of variability contained in the dependent variable (Performance of SMEs) as explained by the independent variable (Training Assistance).

## 2 Experimental design, materials and methods

### 2.1 Data collection

Data was gather from SMEs operators across three states in Nigerian, who have benefited from government training assistance. The study population involves SMEs operators registered under Small and Medium Enterprises Development of Nigeria (SMEDAN), which has a population of 22,971 businesses and 400 business owners were selected as participant for this study. Data were collected based on the copies of questionnaire distributed to SNEs operators. Data was also collected from secondary sources which involve relevant information based on published journals [1], [2], [3], [4], [5], [6], [7], [8]

### 2.2 Data analysis and presentation

Data collected from the questionnaires administered to respondents were analysed and presented as follows. Table 1 indicates the allocation of Copies of Questionnaire, Table 2 shows the reliability statistics, Table 3 shows Model Summary of the effect of Training Assistance on Innovative Performance, Table 4 shows the ANOVA of the effect of Training Assistance on Innovative Performance, Other related literature are can be found in [9], [10].

**Table 1:** Allocation of copies of questionnaire.

**Location**	**Sector**			**Population**	**Proportionate ratio**	**Copies of Questionnaire**
	**Wholesale, Retails & Repairs**	**Manufacturing**	**Agriculture**			
Lagos State	5248	3499	2916	11,663	11,663 ÷ 22971 × 400=203	203

**Table 2:** Reliability statistics.

Cronbach’s Alpha	N of Items
0.876	16

**Table 3:** Model summary of the effect of training assistance on innovative performance.

Model	R	R Square	Adjusted R Square	Std. Error of the Estimate
1	0.578^a^	0.334	0.328	0.46508

a Predictors: (Constant), Training Assistance.

**Table 4:** ANOVA of the effect of training assistance on innovative performance.

Model		Sum of Squares	df	Mean Square	F	Sig.
1	Regression	38.552	3	12.851	59.410	.000^a^
	Residual	77.004	356	.216		
	Total	115.556	359			

^a^Predictors: (Constant), Training participants are helped in diagnosing their own training needs.

b Dependent Variable: innovative performance.

### 2.3 Hypothesis testing

Training assistance has no impact on innovative performance of SMEs in Nigeria.

The model summary table shows how much of the variance of the dependent variable (innovation performance) is explained by the model. In this case, the R square is .334 if expressed by a percentage will be 33.4%. This means that our model explains 33.4% of the variance in the levels of innovation performance.

The F-value is the Mean Square Regression (12.851) divided by the Mean Square Residual (0.216), yielding F=59.410. From the results, the model in this table is statistically significant (Sig=.000) and hence the null hypothesis should be rejected. Therefore, training assistance has impact on innovative performance of SMEs at F_(3,359)_=59.410. Hence, the alternative hypothesis is accepted.

#### 2.3.1 Decision

Thus, based on the result above, it is justified that the null hypothesis be rejected while the alternate hypothesis be accepted. It can therefore be concluded that training assistance has effects on innovative performance of SMEs.

## Acknowledgement

The authors wish to acknowledge the management of Covenant University for providing full sponsorship for this research work.
