# Supplementary material for: Data set on training assistance and the performance of small and medium enterprises in Lagos, Nigeria
Source: Data Brief. 2018 Jul 19;19:2477–80. doi: 10.1016/j.dib.2018.07.023 (PMC6141795; doi:10.1016/j.dib.2018.07.023)
Supplement: Supplementary file 1 — Supplementary material [file mmc1.docx]

The authors of this paper **Data Set on Training Assistance and Performance of SMEs In Lagos, Nigeria declare there is no conflict of interest**

**Fred Peter: Covenant University**

[**fred.peter@covenantuniversity.edu.ng**](mailto:fred.peter@covenantuniversity.edu.ng)

**Aderemi Atayero: Covenant University**

**Odukoya Adedayo: Covenant University**

**Peter Adeshola: Covenant University**

**Maxwell Olokundun:** **Covenant University**

**Adeleke Ogunnaike: Covenant University**

**Olaleke Ogunnaike: Covenant University**

**Stephen Ibidunni: Covenant University**
