# Supplementary material for: Data set on training assistance and the performance of small and medium enterprises in Lagos, Nigeria
Source: Data Brief. 2018 Jul 19;19:2477–80. doi: 10.1016/j.dib.2018.07.023 (PMC6141795; doi:10.1016/j.dib.2018.07.023)
Supplement: Supplementary file 3 — Supplementary material [file mmc3.docx]

**APPENDIX A**

**QUESTIONNAIRE FOR SME OPERATORS**


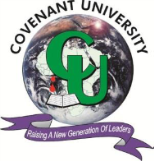


Department of Business Management

College of Business and Social Sciences

Covenant University, Ota, Nigeria

February 10, 2015

Dear Respondent,

**RESEARCH QUESTIONNAIRE**

I am a Ph.D student in the above named institution carrying out a research on **“ Impact of Government Support Programmes on the Performance of SMEs in Nigeria”**. This study is being undertaken in partial fulfilment of the requirements for the award of Ph.D in Business Administration.

Please kindly fill in the correct information needed for the completion of this research. All information supplied will be used for the purpose of this study and will be treated with utmost confidentiality.

Thank you.

Peter, Fred O.

(Researcher)

**RESEACH QUESTIONNAIRE**

**DEPARTMENT OF BUSINESS MANAGEMENT, COVENANT UNIVERSITY, OTA, OGUN STATE**

Dear Respondent,

This questionnaire is based on a Ph.D dissertation exploring “**Impact of Government Support Programmes on the Performance of SMEs in Nigeria**”. I kindly ask for your voluntary co-operation in filling out this questionnaire. All information supplied will be used for the purpose of this study and will be treated with utmost confidentiality.

Thank you

**SECTION A: GENERAL INFORMATION**

**INSTRUCTION:** Kindly tick () as appropriate and comment where necessary.

1. Sex: (a). Male [ ] (b). Female [ ]

2. Age: (a). 18 – 30 [ ] (b). 31 – 40 [ ] (c). 41 – 50 [ ] (d). 51 and above [ ]

3. Highest Educational Qualification: (a). Nil [ ] (b). Primary Sch. [ ] (c). WAEC [ ] ( d). B.Sc. [ ] (e) M.Sc/MBA. [ ] (f) Others (Please specify) _____________________

4. What kind of Business activity are you doing: (a). Manufacturing [ ] (b). Agriculture [ ] (c). Service [ ] ( d). Retail[ ]

5. How old is your Business: (a) 5-9yrs [ ] (b). 10-14yrs [ ] (c). 15 and above [ ]

6. Forms of Business (a). Sole Proprietorship [ ] (b). Family [ ] (c). Partnership [ ] (d).limited liability[ ]

**SECTION B:** Kindly indicate by ticking **()** *as appropriate* whether you “Strongly Agree (SA)”, “Agree (A)”, “Undecided (U)”, “Disagree (D)” or “Strongly Disagree (SD)”.

| S/N | Questions | **SA** | **A** | **U** | **D** | **SD** |
| --- | --- | --- | --- | --- | --- | --- |
|  | **Financial Assistance and Financial Performance** |  |  |  |  |  |
| 1 | SMEDAN facilitate access to adequate credit/loan for the growth of business |  |  |  |  |  |
| 2 | The procedure for obtaining credit/loan assistance is understandable and simple |  |  |  |  |  |
| 3 | The profit of the business has considerably improved over the past 5 year |  |  |  |  |  |
| 4 | The profit margin of the business is satisfactory |  |  |  |  |  |
|  | **Market Assistance and Market Performance** |  |  |  |  |  |
| 5 | SMEDAN provides a range of short courses in the area of marketing, sales promotion and exporting |  |  |  |  |  |
| 6 | SMEDAN did a good job by promoting products/services awareness |  |  |  |  |  |
| 7 | SMEDAN provides on-line platform to improve the visibility of SMEs |  |  |  |  |  |
| 8 | The market share of the business has considerably improved |  |  |  |  |  |
| 9 | The sales turnover of the business is not satisfactory |  |  |  |  |  |
|  | **ADVIS Advisory/Extension Services and Production Performance** |  |  |  |  |  |
| 10 | SMEDAN provides professional information on production management |  |  |  |  |  |
| 11 | SMEDAN provides professional information on product quality, product line and pricing |  |  |  |  |  |
| 12 | Professional information in the area of Accounting, Financing and Book-  Keeping are not adequately provided |  |  |  |  |  |
| 13 | The product/service quality has considerably improved over the past 5 years |  |  |  |  |  |
|  | **TECH Technical/Training Assistance and Innovative Performance** |  |  |  |  |  |
| 14 | SMEDAN provides training and development that is applicable to business |  |  |  |  |  |
| 15 | The training exercises provided are performed by competent professionals |  |  |  |  |  |
| 16 | Supervisors ensure that there is opportunity to use the training immediately |  |  |  |  |  |
| 17 | Supervisors help business to set realistic goals for performing task as a result of training received |  |  |  |  |  |
| 18 | Trainees' performance is measured before, during and after a training program |  |  |  |  |  |
| 19 | Training participants are helped in diagnosing their own training needs |  |  |  |  |  |
| 20 | Appropriate feedback is provided to trainees based on their evaluation results |  |  |  |  |  |
| 21 | The skill, knowledge, & sense of responsibility of the owner has improved |  |  |  |  |  |
| 21 | The quality of product/services has become better |  |  |  |  |  |
| 22 | The work processes have considerably improved |  |  |  |  |  |
|  | **Commitment and SMEs’ Performance** |  |  |  |  |  |
| 23 | Efforts are made to constantly look for new ways to do business better |  |  |  |  |  |
| 24 | I do not feel a ‘strong’ sense of belonging to improve on the business |  |  |  |  |  |
| 25 | I believe in what I do every day to make the business more productive |  |  |  |  |  |
| 26 | I have limited options hence I do not consider leaving this business |  |  |  |  |  |
| 27 | The business suffers when I am under pressure to attend event/party organized by friends/family |  |  |  |  |  |
